# Sulfated Polysaccharides Purified from Two Species of Padina Improve Collagen and Epidermis Formation in the Rat

**Published:** 2013

**Authors:** Moazameh Kordjazi, Bahareh Shabanpour, Ebrahim Zabihi, Mohammad Ali Faramarzi, Farideh Feizi, Hassan Ahmadi Gavlighi, Mohammad Amin Feghhi, Seyed Abbas Hosseini

**Affiliations:** 1 *Department of Fisheries, Gorgan University of Agricultural Sciences and Natural Resources, Gorgan, Iran.*; 2 *Cellular and Molecular Biology research Center (CMBRC), Babol University of Medical Sciences, Babol, Iran.*; 3 *Department of Physiology & Pharmacology, Babol University of Medical Sciences, Babol, Iran.*; 4 *Department of Pharmaceutical Biotechnology, Tehran University of Medical Sciences, Tehran, Iran. *; 5 *Bioprocess Engineering Center, Technical University of Denmark, Lyngby, Denmark.*; 6 *Persian Gulf Biotechnology Park, Persian Gulf Biotechnology Research Center, Qeshm Island, Iran.*

**Keywords:** Brown algae, fucoidan, wound healing

## Abstract

Sulfated polysaccharides have shown promising effects on wound healing processes along with many other biological activities. The sulfated polysaccharides extracted from two algae species habitats in Persian Gulf were studied *in vivo* for their effects on collagen formation and epidermal regeneration. The polysaccharides were purified from aqueous extracts of *P. tetrastromatica* and *P. boergesenii* using CaCl2 and ethanol precipitation. The sulfate content of each polysaccharide was determined. Two identical wounds (either burn or excision) were made on the back of 4 groups of male Wistar rats (10 rats per group) under anesthesia. The algal polysaccharide ointments (2%) were applied twice daily on one side and the other wound was treated with Eucerin (as control). The rats were sacrificed on day 7 or 14, and then the wound samples were examined for epidermal thickness by light microscope. Furthermore, hydroxyproline content (as a marker of collagen formation) was spectro-photometrically measured. The polysaccharides purified from *P. boergesenii* had higher sulfate content (32.6±1%) compared to *P. tetrastromatica* (19±1%). Both algal polysaccharides showed some improvements in collagen formation (hydroxyproline content) and epidermal thickness in both wound models compared to the vehicle. The sulfated polysaccharides purified from *P. tetrastromatica* and *P. boergesenii* seaweeds are able to induce collagen formation and epidermal regeneration in the two wound models. The superior healing properties of *P. boergesenii* polysaccharides might be correlated to its higher sulfate content. Both algal polysaccharides are good candidates for wound healing clinical trials.

Marine algae have been introduced as important sources of pharmacologically active molecules since last century. Polysaccharides derivatives, along with other chemically active compounds, constitute the major component of macroalgae (seaweeds). An important fucose containing sulfated polysaccharide isolated from different species of brown algae is called fucoidan. The fucoidans have been extensively studied and have shown various pharmacological activities including anticoagulant/antithrombotic, antivirus, antitumor, and anti-inflammatory effects among many other biological activities. In order to develop new therapeutics or functional foods, fucoidans have been subjected to numerous studies in recent years (1). Compared to animal sulfated polysaccharides (e.g. heparin, chondroitin sulfate…), fucoidans are widely available from cheaper sources.

The chemical composition and structure of fucoidans are highly influenced by the algae species, habitat, harvesting time, and their environmental conditions (2). These polysac-charides are composed of different proportions of monosaccharides such as fucose (the main monomer), uronic acid, galactose, xylose and sulfate (1, 3). Bioactive sulfated polysaccharides (e.g. heparan) have shown positive changes in animal cells paracrine interactions in a way that could benefit in wound healing processes.

A search for new methods of wound healing enhancement has been always one of the subjects of interest to medical scientists and fucoidans are among those natural substances that had highly attracted biological investigations in the last few decades.

Dermal wounds repair is a complicated process which require quick reconstruction of the skin structures (4-5). As the most important consti-tuent of mammalian dermis, efficient collagen formation is a key factor to dermal wound repair.

In the northern coastal area of Persian Gulf, especially Qeshm Island, there are many brown algae species from different genera like Padina, Cystoseira, Sargassum which contain potentially bioactive substances and have not yet been studied enough. In the current study we aimed to extract the sulfated polysaccharides (fucoidans) from two species of genus Padina habitat to this region and to evaluate their effects on epidermis and collagen formation in two wound types.

## Materials and Methods

**Algae samples**

Fresh brown seaweeds containing *P. tetrastr-omatica* and *P. boergesenii* from Qeshm Island were collected manually from the intertidal zone at Kani (55º23 765 E and 26º34 344 N) and Bahman port (56º16 646 E and 26º57 645 N) in February 2011. The samples were thoroughly rinsed with fresh water to eliminate foreign materials such as sand, shells, their hold-fasts and epiphytes. The voucher specimens are deposited in “Persian Gulf Biotechnology Park” (*P. tetrastromatica* No: 66-20p and *P. boergesenii *No: 44-14p). Dried algal mass was powdered and sieved through a mesh size of 1 mm and kept at -20ºC in plastic containers.

**Algae extraction and ointment preparation**

Extraction with hot water and consequent alginate precipitating with CaCl_2_ (Sigma-Aldrich, UK) was used for the extraction of sulfated polysaccharides from the two algae samples. Then the fucoidan polysaccharides were separatedfrom the other components by alcohol (70%) precipitation according to the method of Yanget al. (6). Using Eucerin® and minimal distilled water, 2% ointments of each hydrophilic extract was prepared.

**Determination of sulfate content**

The sulfate contents of the two polysaccharide samples, as a marker of fucoidan, were determined by the method of Jackson and Mccandless (7) using agarose-BaCl_2_ reagent (0.01% and 0.5%). The Na_2_SO_4_ was used as calibration standard.

**Animals and experimental wounds**

Forty male Wistar rats (200-250 g) were housed under standard conditions of temperature, 12 hours light/dark with food and water *ad libitum*. Animal maintenance and care complied with the current laws of Babol University of Medical Sciences. They were acclimatized to laboratory conditions at least 24 hours before conducting the experiments.

The animals were divided randomly into 4 groups of 10 and under anesthesia with pentobarbital (50 mg kg, i.p.), two symmetrical wounds were made on the back of a shaved rats. To induce burn wounds, a soldering iron (10 mm) preheated up to 100°C was contacted to depilated skin of rats for 20 sec. on both sides (right: treatment, left: control) according to the method of Kimura with minor modifications (8). Also in another group of rats, open excision type of wounds of a standard size (4 cm^2^) was created as described by Raghavan et al. (9). Then the animals were kept in separate cages and wounds were monitored during the next 14 consecutive days while 250 mg of each ointment were applied on the wounds every day (5 days a week, twice a day). Eurecin was used as negative control on each rat’s left side wound.

**Measurment of epidermis thickness**

On the days 7 and 14 post treatment, five rats of each group were sacrificed and autopsy skin samples were fixed in a 10% buffered formal-dehyde solution. The paraffin-embedded sample blocks were sectioned in 5 mm increments. The sections were perpendicular to the anterior-

posterior axis and perpendicular to the surface of the wounds. The sections were mounted on a glass slide and stained with haematoxylin-eosin or Van Gieson. The slides were examined with an Olympus BX41 light microscope (Olympus Corporation, Tokyo, Japan). For each sample, five serial sections from different parts of the paraffin block were photographed with a digital camera (Canon SX 230 HS). Ten measurements were performed per section using “Motic Images Plus” software (Motic Co, Xiamen, China).

**Hydroxyproline assay**

**Fig. 1:**
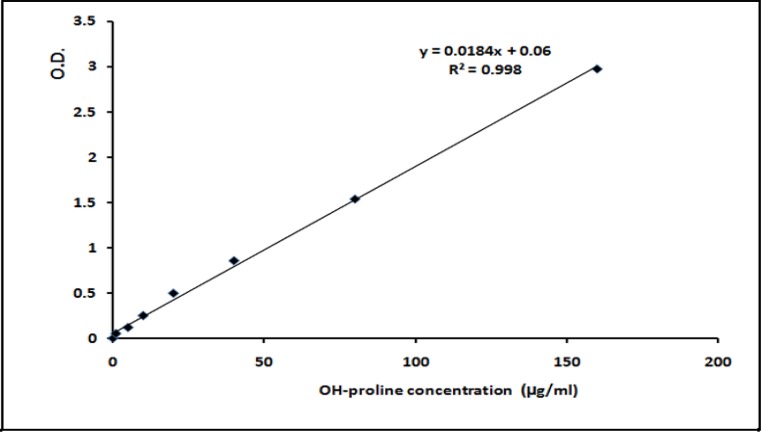
Calibration curve for spectrophtometric analysis of tissue hydroxyproline (OH-prolin) content

All chemicals and reagents were purchased from Sigma-Aldrich Co. (United Kingdom). The hydroxyproline content of granulation tissues was measured as a marker of collagen formation according to the methods of Udenfriend (1960) and Switzer and Summer (1971) with minor modifica-tions (10-11). Briefly, after hydrolyzing the skin samples in HCl 6 N at 105ºC for 14-16 hrs, the produced hydroxyproline was oxidized by Chlora-mine-T (1.4% w/v in acetate-citrate buffer, pH 6.0) and incubated for 20 min in Ehrlich`s reagent at 60ºC. After acidic extraction by toluene, the optical density of the solution was read at 543 nm using a Beckman DU-600 spectrophotometer (Beckman-coulter Co; USA). The hydroxyproline content of each sample was calculated using a standard curve which is linear in the range of 1-200 µg/ml (Fig. 1).

**Statistical Analysis**

The results were expressed as mean±standard error (S.E.). All statistical analyses were performed using the SPSS software package, version 18 (SPSS Inc, Chicago). Student’s paired or independent *t*-test was used to test significance of difference between means. P < 0.05 was considered as signi-ficant difference between means.

## Results

**Polysaccharides sulfate content**

The sulfated polysaccharides extraction yield for *P. tetrastromatica* and *P. boergesenii* were 1±0.5% and 4.5±0.5% and their sulfate contents were 19±1% and 32.6±1% respectively.

**Epidermal thickness**

**Fig. 2:**
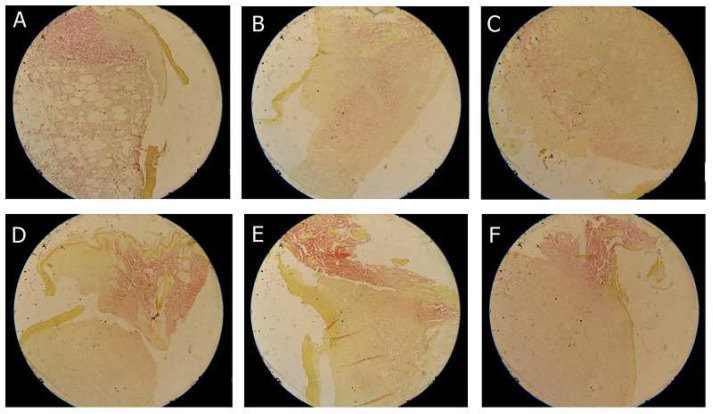
Epithelialization and collagen formation in burn (A, B, C) and excisional (D, E, F) wounds, 14 days after treatment by either ointment vehicle (A, D) or sulphated polysaccharides extracted from *P. boergesenii* (B, E) and *P. tetrastromatica *(C, F). Van Gieson stained slides shows higher collagen formation (red spots) and epithelialization of the extracts (P. tetrastromatica and P. boergesenii) and the negative control (ointment base treated) groups.

The epidermal thickness at different time points after inducing burn or excision wounds are presented in Tables 1 and 2 respectively (Fig. 2). 

**Table 1:** Comparison of epidermis thickness (µm) (mean±SE) in burn wound

**Group**	** Day**	
	** 7**	** 14**
***P. tetrastromatica***	46±18	59±18
**Control**	28±11	30±5
***P. boergesenii***	32±9	56±19
**Control**	19±10	31±10

**Table 2:** Comparison of epidermis thickness (µm) (mean±SE) in excisional wound

**Group**		** Day**	
		**7**	** 14**
***P. tetrastromatica***	19±1*		14±4
**Control**	8±2		11±6
***P. boergesenii***	24±7*		17±4
**Control**	12±4		10±2

* Significant difference (P < 0.05) compared to the vehicle treated wound on the same animal.

**Hydroxyproline content**

The collagen formation (measured as the hydroxyproline content of wound) was significantly higher than control in *P. boergesenii* treated burn wounds at day 14 (Table 3). While the sulfated polysaccharides extracted from both *P. boergesenii *and* P. tetrastromatica *were significantly efficient at day 7 in excisional wounds (P < 0.01) (Table 4).

## Discussion

Both purified polysaccharides from *P. tetrastromatica* and *P. boergesenii* are able to improve collagen formation (hydroxyproline content) and significantly increase epidermal thickness in rat excisional wound model. The sulfated polysaccharides of *P. boergesenii* showed higher activity compared to *P. tetrastromatica* in burn (Table 3) and excisional wound models (Tables 2 and 4) which is in concordance with its higher sulfate content. Sulfated polysaccharides are important bioactive substances obtained from marine algae including Phaeophyceae (12). An important category of these sulfated polysa-ccharides include fucoidans with a broad range of pharmacological activities. Anticoagulant, anti-inflammatory and antioxidant activities are only few examples in the long list of fucoidans’ effects (1, 13). The complex structure of fucoidan polymers and the extent of sulfation have great impact on their biological activities (13-14). On the other hand, it has been well documented that the biocomposition of fucoidans is highly influenced by the growing seaweeds environment and the season of harvest (2, 15).

**Table 3:** Comparison of hydroxyproline (mg/g) (mean±SE) in burn wound

**Group**	** Day**	
	** 7**	** 14**
***P. tetrastromatica***	27.7±0.1	18.1±0.6
**Control**	14.5±0.1	11.9±0.7
***P. boergesenii***	24.8±3.1	26.9±2.1**
**Control**	24.6±3.2	17.1±0.6

** Significant difference (P < 0.01) compared to the vehicle treated wound on the same animal.

**Table 4:** Comparison of hydroxyproline (mg/g) (mean±SE) in excisional wound

**Group**	** Day**			
	** 7**			**14**
***P. tetrastromatica***		29.1±0.7**	24.6±2.2	
**Control**		21.8±0.5	21.2±1.8	
***P. boergesenii***		41±0.2**	35.3±4.4	
**Control**		22.6±0.3	22.4±0.6	

** Significant difference (P < 0.01) compared to the vehicle treated wound on the same animal.

In the current study, the fucoidan sulfated polysaccharides purified from two brown algae (*P. tetrastromatica* and *P. boergesenii*) habitat in Persian Gulf, were evaluated for their wound healing properties for the first time.

**Fig. 3:**
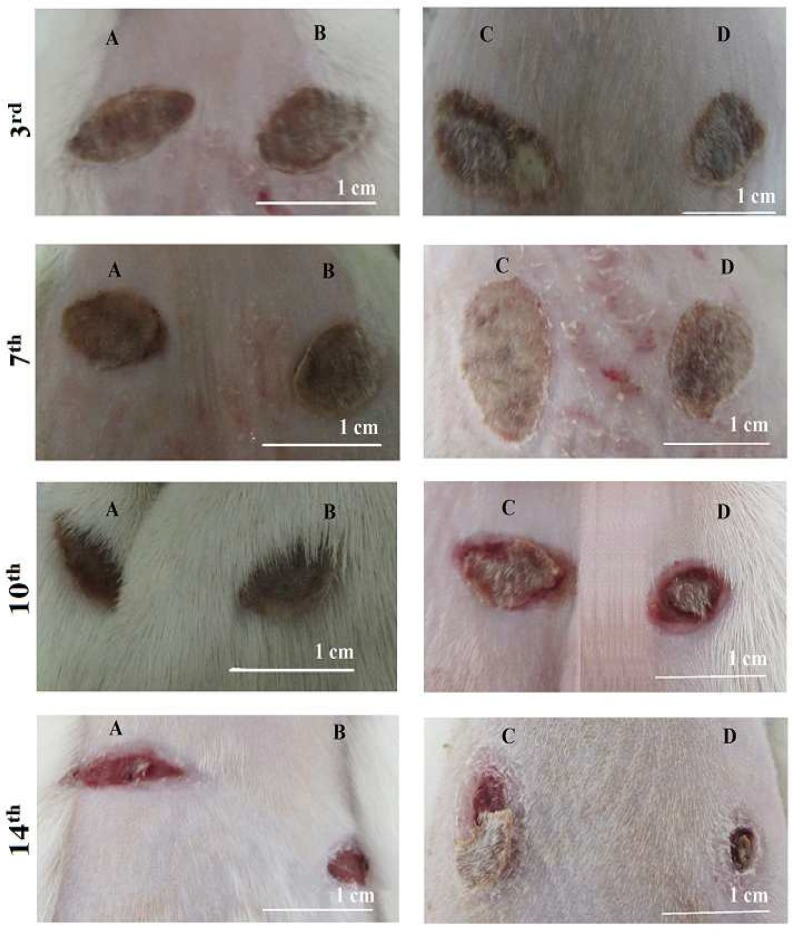
The photographs of burn wounds treated by two different algal extract ointments on different days after treat-ment. A: Control wound corresponding to *P. tetrastromatica;* B: Wound treated by *P. tetrastromatica*; C: Control wound corresponding to *P. boergesenii*; D: Wound treated by *P. boergesenii*. The *P. boergesenii* extract ointment shows better wound healing properties compared to *P. tetrastromatica* (B). This effect is more obvious at the day 14^th^ after burn

**Fig. 4:**
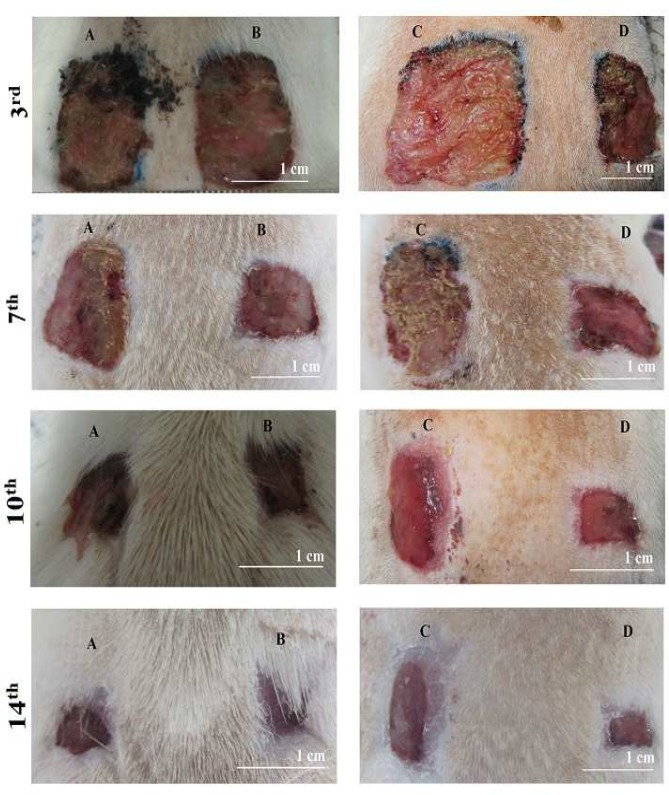
The photographs of excisional wounds treated by two different algal extract ointments on different days after treat-ment. A: Control wound corresponding to *P. tetrastromatica;* B: Wound treated by *P. tetrastromatica*; C: Control wound corresponding to P. boergesenii; D: Wound treated by P. boergesenii. Both algal extract ointments (B, D) have signifi-cant effects on wound healing compared to the control (A, C). The healing effect is obvious from day 7^th^ after excision

Since the polysaccharides method of extraction/purification was specific to fucoidans, it could be assumed that the sulfated polysaccharides purified by this procedure mostly belong to fucoidans. The sulfate content of *P. boergesenii* extracted polysaccharides (32.6±1%) was quite higher than *P. tetrastromatica* (19±1%). The higher sulfate content might be attributed to the better wound healing properties (13, 16). Eventually the better healing effects of *P. boergesenii* on cellular and molecular changes in burn or excision wounds could be observed even by gross daily examination of the wounds (Fig. 3, 4). 

From a mechanistic point of view, a different model of wounds (e.g., burn, excisional, incisional, hypoxic…) could be used to evaluate new candidate agents. While none of the two extracted polysaccharides showed significant effect on epidermal thickness in burn wounds, both have increased the newly formed epidermis thickness at the day 7 in excisoinal wound model which might reflect the differences in the healing processes of the two wound types. On the other hand, the skin hydroxyproline content as a marker of collagen formation by fibroblasts, which is a critical step for wound closure and healing processes (17), has increased by both extracted polysaccharides in excisional wound model (at day 7) (Table 4) (Fig. 2). In burn wound, this increase in tissue hydroxyproline content was significant only for *P. boergesenii* (at day 14) (Table 3) which might reflect the higher efficacy of *P. boergesenii *and could be related to its higher sulfate content.

Stimulation of fibroblast proliferation and improvement in paracrine cellular interactions have been proposed as two mechanisms for polysac-charides wound healing properties (17-18). Mesen-chymal stem cells recruitment into wounded tissues and their transdifferentiation into different type of skin cells might be another explanation for efficacy of the two sulfated polysaccharides (19). This phenomenon ultimately results in higher collagen formation and increases epidermal thickness. The increased collagen synthesis together with accelerated epidermis regeneration observed in this study appeared to be in concordance with those reported earlier (18).

As a conclusion, the sulfated polysaccharides from *P. tetrastromatica* and *P. boergesenii*, two brown algae species habitat  in northern coasts area of Persian Gulf, have significant wound healing effects in rat’s burn and excisional wound models. The polysaccharides purified from P. *boergesenii* have higher sulfate content and seem to be more potent than *P. tetrastromatica* especially in excisional wound model.
